# The Compounding Effect of Rurality on Health Disparities Among Black Patients with COVID-19

**DOI:** 10.13023/jah.0304.03

**Published:** 2021-10-25

**Authors:** Jessica E. Johnson, Ruchi Bhandari, Allison Lastinger, Rebecca Reece

**Affiliations:** Department of Medicine, Division of Infectious Diseases, West Virginia University, Morgantown WV; Department of Epidemiology, West Virginia University, Morgantown WV; Department of Medicine, Division of Infectious Diseases, West Virginia University, Morgantown WV; Department of Medicine, Division of Infectious Diseases, West Virginia University, Morgantown WV

**Keywords:** Appalachia, COVID-19, racial disparities, rural America, West Virginia

## Abstract

**Background:**

West Virginia had garnered national attention for its vaccination rollout against coronavirus 2019 (COVID-19). Outcomes of this mostly rural population, however, have been underreported. As the pandemic continues, identifying high risk populations remains important to further epidemiologic information and target vaccines.

**Purpose:**

The objective of this study is to examine the effects of COVID-19 and the influence of race and rurality on hospitalization and outcomes in Appalachians.

**Methods:**

In this retrospective study, data from patients who tested positive and were admitted for COVID-19 and seen within the state’s largest health system (West Virginia University Health System) between March 18 and September 16, 2020 were analyzed. Cases were stratified into rural or urban based on rural urban continuum codes (RUCCs) and by race into ‘white,’ ‘black,’ or ‘other.’ Associations between rurality, rurality and race, and outcomes were assessed.

**Results:**

A total of 2011 adult West Virginians tested positive, of which 8.2% were hospitalized. Of the hospitalized patients, 33.5% were rural and 11.6% were black. Rural black patients were three times more likely (OR: 3.33; 95%CI:1.46–7.60) to be admitted. Rural blacks were also more likely to have a history of obstructive pulmonary disease (OR: 2.73; 1.24–6.01), hypertension (OR: 2.78; 1.38–5.57), and multiple chronic conditions (3.04; 1.48–6.22).

**Implications:**

Rural blacks were more likely to have risk factors for severe COVID-19 influencing their increased risk of hospitalization. These findings support that race as a risk factor for severe COVID-19 is compounded by rurality and identifies an important target group for vaccination.

## INTRODUCTION

Research of the clinical characteristics and outcomes of hospitalized patients with coronavirus disease 2019 (COVID-19) remains an important area of study. This information aids in understanding the compounding risk factors of severe acute respiratory syndrome coronavirus 2 (SARS-CoV-2), the virus that causes COVID-19, and its effects on different populations. This work helps improve treatment and identify vaccination target groups for public health workers.

Since the spread of the pandemic to the U.S., chronic conditions including diabetes, hypertension, obesity, chronic lung disease, and cardiovascular disease as well as a history of smoking have all been identified as independent risk factors for severe disease.^1^–^3^ Many studies have also highlighted racial and ethnic disparities in the U.S. with minorities disproportionately affected by COVID-19. This is especially true amongst black Americans.^4^

Prior to the SARS-CoV-2 pandemic, population health research had found an increasing divergence between American rural and urban mortality rates. This included a lag for rural blacks and rural whites compared to their same-race urban counterparts with urban whites having overall the best outcomes.^5^ This discrepancy is influenced by increased rates of chronic illnesses,^6^ smoking,^7^ obesity,^8^ and limitations to health care access seen in rural areas.^9^

The influence of rurality is important when studying the health of the southern United States. According to the U.S. Census Bureau, 60 million people live in nonmetropolitan areas with 47.6% of those in the southern region. Of the southern states, West Virginia has the highest percent of its population living in rural areas at 50.9% ranking third nationally behind Vermont and Maine.^10^ West Virginia is also the only state entirely a part of Appalachia.^11^

The effects of Appalachian rurality are demonstrated in West Virginia, which has some of the highest rates of obesity, diabetes, cardiovascular disease, chronic obstructive pulmonary disease (COPD), and smoking in the country.^12^,^13^ West Virginia also has an overall older population with over a third of its residents living with multiple chronic conditions (MCC), which the Centers for Disease Control and Prevention (CDC) defines as having two or more of the following: arthritis, asthma, COPD, cancer, cardiovascular disease, hepatitis, hypertension, stroke, or kidney failure.^14^ MCC is another risk factor for progression of COVID-19 disease.^2^,^14^

The combined effects of rurality and race on COVID-19 outcomes remains underreported. Recent analyses suggest an unequal distribution with higher death rates seen in rural areas with large minority populations.^15^,^16^ These populations studies, however, are subject to the ecological fallacy and cannot directly infer if deaths from COVID-19 were among minorities or just in areas where more minorities lived. The objective of this study is to increase this knowledge and examine individual risk to see if race, rurality, or a combination of race and rurality are responsible for these findings. Herein is presented the only study to date to compare baseline chronic conditions and clinical outcomes of the first positive cases of SARS-CoV-2 and hospitalizations for COVID-19 disease within West Virginia. The existence of health disparities in this population was assessed by looking at demographic variables including rurality and race. This information will also outline the start of the COVID-19 pandemic in West Virginia, almost a year prior to widespread availability of the vaccine.

## METHODS

### Study design and participants

This retrospective, observational study included cases of confirmed SARS-CoV-2 by positive qualitative polymerase-chain-reaction (PCR) assay from March 18, 2020 to September 16, 2020, the date of data extraction. Using the multicenter research network TriNETX (Cambridge MA), deidentified, real time electronic health records of all persons with positive SARS-CoV-2 PCR assays at any hospital or testing site within the West Virginia University (WVU) Health System were collected. This included those who were hospitalized secondary to COVID-19 disease. WVU Health System, based in Morgantown, West Virginia, is the largest health system in the state and includes 16 managed hospitals, two affiliated hospitals, and five institutes.^17^ Cases who were not West Virginian residents were excluded as well as those <18 years as no hospitalizations in this age group had occurred.

### Data collection

Data extracted for all cases included demographic characteristics (age, gender, patient reported race and ethnicity, and ZIP code). ZIP codes were used to determine whether a patient lived in a rural or urban area based on the U.S. Department of Agriculture rural–urban continuum codes (RUCCs) which defines county level degree of urbanization and has been used as a measure of rurality in previous studies.^5^,^18^ Cases were defined as either urban (RUCCs 0–3) or rural (RUCCs 4–9). Based on this classification, 21 counties in West Virginia are considered urban and 34 are considered rural.

Racial categories were grouped into ‘white’, ‘black’, or ‘other or unknown’. Ethnic categories were grouped into ‘Hispanic’, ‘non-Hispanic’, or ‘unknown’. All white and black cases reported their ethnicity as non-Hispanic. Other variables collected included the most recent body mass index (BMI, weight in kilograms divided by the height in meters squared) recorded within the past 12 months of data extraction. Underlying chronic conditions were assessed using International Classification of Diseases, 10th revision [ICD-10] diagnosis codes linked in ambulatory, specialty, and/or inpatient encounters. ICD-10 codes used included: I10–15 for hypertension; J41–45 for asthma and/or COPD; and I20–25 and I63–73 for cardiovascular disease. The number of these diagnoses was totaled for each case and divided into low risk (0 or 1 chronic condition) and high risk (≥2 chronic conditions). Smoking history was also assessed using ICD-10 code F17 as well as Z87.891, Z71.6, and/or Z72.0.

Additional variables were gathered for patients hospitalized for COVID-19 disease including need for care in the intensive care unit (ICU), and/or need for mechanical ventilation. Patients included had completed initial admission for COVID-19 at data retrieval and were not currently still admitted for any reason. Reported death anytime during the study period was collected.

### Statistical analysis

Characteristics of adult West Virginians with SARS-CoV-2 were compared based on clinical characteristics, rurality (rural vs. urban based on RUCCs), and race (white, black, or other). Characteristics of all positive cases are summarized in Table 1 (2011 cases). Descriptive statistics were calculated for all variables. Chi square test of independence examined the association between categorical variables. Categorical measures are presented as frequencies and percentages. Continuous measures are presented as means and standard deviations.

Bivariate logistic regression analyses were used to calculate the unadjusted odds ratio and 95% confidence interval (CI) for three associations of (1) Hospital admission status with demographics, chronic conditions, and clinical outcomes (Table 1); (2) Rurality status with demographics, chronic conditions, and clinical outcomes (Table 2); and (3) Combined race and rurality status with demographics, chronic conditions, and clinical outcomes (Table 3). For the associations presented in Table 3, a variable was created by combining race and rurality status, resulting in six categories, namely, rural white, rural black, rural other, urban white, urban black, and urban other. All analyses were done in IBM SPSS v.26.

## RESULTS

### Characteristics and outcomes of West Virginians with SARS-CoV-2

Table 1 presents the characteristics of the entire sample population and those hospitalized with COVID-19 disease. A total of 2168 West Virginians tested positive for SARS-CoV-2 of whom 157 were <18 years and excluded from statistical analysis. Of the remaining 2011, the mean age was 44 years (range 18–96), most were female (51.6%), white (80.2%), and non-Hispanic (84.5%). Based on RUCCs, 34.4% of cases were rural.

### Characteristics and outcomes of West Virginians hospitalized with COVID-19

Of the 2011 West Virginians who tested positive, 8.2% were hospitalized for COVID-19 disease. Hospitalized cases were mostly white (79.9%), non-Hispanic (93.9%), and >65 years (56.7%) with a mean age of 66 years (range 19 to 96). Over a third (33.5%) were from rural areas. Black patients comprised 11.6% of admissions. In terms of complications, 41.0% of hospitalized patients required care in the ICU, 25.0% required mechanical ventilation, and 20.7% died over the study period.

### Association of hospitalization

Table 1 also presents the bivariate association of hospitalized and non-hospitalized cases with demographic and clinical characteristics. Compared with patients who were not admitted, hospitalized patients were twice as likely to be black compared to white (OR: 1.99; 95%CI: 1.19–3.35); more likely to be older with reference to patients younger than 40 [40 and 64 years (OR: 5.95; 95%CI: 3.29–10.77) and 65 and older (OR: 26.80; 95%CI: 15.01–47.84)]; more likely to have a smoking history (OR: 4.03; 95%CI: 2.86–5.68); more likely to have 2 or more chronic conditions (OR: 17.81; 95%CI: 12.39–25.62); and more likely to die (OR: 39.99; 95%CI: 0.28–0.94).

### Association of rurality

Table 2 presents the bivariate association between rurality status and various characteristics of all sample patients. Compared to whites, blacks (OR: 1.59; 95%CI: 1.06–2.40) and people from other races (OR: 1.56; 95%CI: 1.17–2.08) were more likely to live in urban areas. Compared to non-Hispanics, Hispanics were more likely to live in urban areas (OR: 14.01; 95%CI: 4.40–44.56). Compared to people in the older age group (65 years), those between 40 and 64 years (OR: 1.34; 95%CI: 1.03–1.75) and <40 years of age (OR: 1.74; 95%CI: 1.34–2.25) were more likely to live in urban areas. The associations of rurality with gender, obesity, chronic conditions, smoking, hospitalization, and mortality were not statistically significant.

### Association of race & rurality

Table 3 presents the bivariate association between combined race–rurality status and various patient characteristics. Compared to urban whites, rural blacks were more likely to be admitted to the hospital (OR 3.33; 95%CI: 1.46–7.60); have a history of hypertension (OR: 2.78; 95%CI: 1.38–5.57); obstructive pulmonary disease (OR 2.73; 95%CI: 1.24–6.01); and have 2 or more chronic conditions (OR 3.04; 95%CI: 1.48–6.22). Compared with urban whites, rural others and urban others were more likely to be male and less likely to report hypertension, cardiovascular disease, asthma, and diabetes. They were also less likely to have a history of smoking.

## DISCUSSION

This study examines the characteristics and clinical outcomes of a large cohort of SARS-CoV-2 cases in West Virginia. Although only 3.6% of West Virginia’s population is black,^11^ in this study 6.2% of those who tested positive for SARS-CoV-2 and 11.5% of those admitted for COVID-19 disease were black. Hospitalized patients were twice as likely to be black compared to white. Rural cases were more likely to be older; however, rurality alone was not associated with an increased risk of hospitalization or death. When considering rurality and race, however, rural black West Virginians were more likely to be admitted and have a history of hypertension, obstructive pulmonary disease, and multiple chronic conditions.

Health disparities among the black population in the U.S. have been highlighted during the COVID-19 pandemic. Over representation of black cases and admissions for COVID-19 have been demonstrated in other largely rural, southern states including Louisiana and Georgia.^4^,^19^ Multiple factors in West Virginia likely contribute to these findings in our state’s black population.

One risk factor may have been living in a county where the virus was more prevalent at the start of the pandemic. Two neighboring counties, Berkeley and Jefferson, represented close to thirty percent of all positive cases in this study. Some of the first confirmed cases of SARS-CoV-2 in the state were from these counties.^20^ This is likely secondary to their large commuter population due to their proximity to metropolitan areas including Baltimore MD, and Washington D.C. These counties also have larger black populations (with both at 7.9%) compared to other areas of West Virginia.^21^ Both counties are considered urban based on RUCCs and did not contribute to the number of rural black hospitalizations.

Differences in jobs between white and black West Virginians may also contribute to an increased risk of viral exposure. According to a 2020 report by the West Virginia Center of Budget and Policy, one-third of black workers versus one-fifth of white workers are employed in the service industry.^22^ Black West Virginians are also twice as likely to be living in poverty with households averaging thirty percent less than that of white West Virginians.^22^ This can lead to crowding allowing for easier spread of respiratory pathogens.

Underlying chronic conditions have been shown to increase the risk of developing severe COVID-19 disease.^1^–^3^ Compared to other states, West Virginia ranks as the highest or second highest in prevalence of obesity, diabetes, COPD, and heart disease.^13^ Many of these conditions especially effect the health of West Virginia’s black population. For instance, according to the CDC, 37.7% of all adult West Virginians are obese. This percent is even higher at 47.7% among the black population.^12^ Death rates from diabetic related complications and heart disease are also almost twice as high in black West Virginians compared to white.^22^

In this study, rurality as a risk factor for hospitalization from COVID-19 disease was demonstrated in black cases. While most black West Virginians live in urban areas,^11^ 14.6% of rural admissions identified as black. Population studies have demonstrated that death rates from COVID-19 are higher among rural counties with larger shares of minority populations, including black patients.^15^ This is likely due to the compounding effect rurality has on many racial discrepancies. Rural communities have higher prevalence of obesity, diabetes, hypertension, and cardiovascular disease which are already higher among the black population.^23^–^26^

At the start of the SARS-CoV-2 vaccine rollout, West Virginia was applauded for its initial success. By the end of January 2021, the state had administered over eighty percent of its allocated vaccine doses and was considered the best in the nation. This was secondary to the state’s use of local pharmacies and the National Guard to administer vaccines quickly and effectively.^27^

As of January 29, 2021, reports from the Kaiser Family Foundation (KFF) noted black Americans were being vaccinated at lower rates than white in many states. This included West Virginia where vaccination rates for blacks were 6.2% versus 11.9% for whites.^28^ A follow up study by KFF that analyzed rates to June 14, 2021 noted improvement with 39% of black and 43% of white West Virginians receiving at least one COVID-19 vaccine.^29^ While this data is encouraging, it is unknown if this represents improved vaccination allocation or a peak in residents willing to be vaccinated.

Like other southern states, West Virginia has now fallen behind the national percent in vaccinated residents. As of June 21, 2021, the CDC reported that 53.5% of the eligible population in the U.S. and only 41.3% of West Virginia’s had received at least one dose of vaccine.^30^

West Virginia’s state motto is *montani semper liberi* which translates to ‘mountaineers are always free’.^31^ As West Virginia remains vulnerable to the effect of the COVID-19 pandemic, it must continue to improve its vaccination rates to ensure this freedom can continue. This includes interventions to ensure rural black West Virginians are being vaccinated against COVID-19. Such initiatives could include utilizing trusted community-based organizations to help educate and vaccinate rural black populations. Health care providers must acknowledge vaccine hesitancy seen in black Americans related to medical mistrust rooted in discrimination. Providers must be educated on how to understand these beliefs and still encourage vaccination without appearing confrontational or dismissive. Local and state health departments should ensure that authentic information about vaccines is being distributed that is culturally competent. Implementing community and place-based approaches to ensure equitable COVID-19 vaccine access is also important in rural communities to decrease barriers to reach at risk populations.

## IMPLICATIONS

In this study, rural black West Virginians were found to have higher rates of risk factors for severe COVID-19 influencing their increased risk of hospitalization. This makes them especially important to target by ongoing vaccination efforts. This study also identifies the need for additional research on the effects of the COVID-19 pandemic on rural minorities in other areas of Appalachia. The epidemiological information obtained also shows how a mostly rural, Appalachian states remains vulnerable to COVID-19 due to its high rates of underlying chronic conditions, obesity, and smoking. As vaccination rates in West Virginia appear to be stalling, this may help encourage all those eligible to get vaccinated.

### Limitations

The findings of this study should be interpreted with several limitations in mind. First, this analysis is inherently limited because of its retrospective nature. Also, rural–urban status is multidimensional. Here, RUCCs were used as a marker for rurality, which defines degree of urbanization as the county level. Counties themselves, however, can be heterogenous with this not accurately representing findings on a smaller geographic level. Fatality and hospitalization rates also reflect time before more treatment options were available as well as the COVID-19 vaccine. This study only included positive cases from the WVU Health System and does not reflect viral prevalence in areas where affiliated hospitals are not located. Finally, this study relied on structured data captured in electronic medical records and uploaded into TriNetx. Study findings are therefore subject to the accuracy and completeness of imputed data. Using BMI of less than 30 to denote ‘normal weight’ may have missed the contributing significance of being underweight/malnourished defined as a BMI of ≤18.5. Notwithstanding these limitations, this study provides comparative epidemiologic characteristics of West Virginians, and the differences seen based on rurality and race among COVID-19 cases which is underrepresented in the medical literature to date.

**SUMMARY BOX**

**What is already known on this topic?**

The current pandemic has highlighted racial disparities in many areas of the U.S. with black Americans disproportionately affected by COVID-19. The combined effect of rurality and race on COVID-19 outcomes remains underreported.

**What is added by this report?**

This study examined the intersection between race and rurality during the COVID-19 pandemic in West Virginia. Here, rural black cases were three times more likely to be hospitalized for COVID-19 disease. Rural black patients were found to have higher rates of risk factors for severe COVID-19 disease including hypertension, obstructive pulmonary disease, and multiple comorbidities.

**What are the implications for future research?**

These findings suggest that the race penalty of COVID-19 is worsened by rurality. Future research would help delineate if this is true in other areas of Appalachia. This research would help identify high risk groups in other areas who should be targeted by vaccine efforts.

## Figures and Tables

**Table 1:** Association between hospitalization and clinical characteristics of adult West Virginians with SARS-CoV-2*

	All Cases		Hospitalized				
VARIABLES	(N = 2011)		(N = 164)		OR	(95%CI)	*P value*
RUCCs							
Rural	692	(34.4)	55	(33.5)	Ref.		…
Urban	1319	(65.6)	109	(66.5)	1.04	0.74–1.46	0.81
Age group year – no (%)							
18 – <40	970	(48.2)	14	(8.5)	Ref.		…
40 – 65	711	(35.4)	57	(34.8)	**5.95**	**(3.29–10.77)**	**<0.001**
≥65	330	(16.1)	93	(56.7)	**26.80**	**(15.01–47.84)**	**< 0.001**
Gender – no (%)							
Male	964	(47.9)	83	(50.6)	Ref.		…
Female	1038	(51.6)	81	(49.4)	0.92	(0.67–1.27)	0.62
Race – no (%)							
White	1613	(80.2)	131	(79.9)	Ref.		…
Black	125	(6.2)	19	(11.6)	**1.99**	**(1.19–3.35)**	**0.01**
Other or Unknown	273	(13.6)	12	(7.3)	**0.51**	**(0.28–0.94)**	**0.03**
Ethnicity – no (%)							
Non-Hispanic	1699	(84.5)	154	(93.9)	Ref.		…
Hispanic	82	(4.1)	8	(4.9)	1.08	(0.51–2.28)	0.85
Obesity – no (%)							
Obese – no	978	(48.6)	73	(44.5)	Ref.		…
Obese – *yes*	763	(37.9)	91	(55.5)	1.08	(0.51–2.28)	0.85
Smoking history – no (%)							
Smoker – no	1710	(85.0)	102	(62.2)	Ref.		…
Smoker – yes	301	(15.0)	62	(37.8)	**4.03**	**(2.86–5.68)**	**< 0.001**
Chronic Conditions – no (%)							
Hypertension – no	1548	(77.0)	35	(21.3)	Ref.		…
Hypertension – *yes*	463	(23.0)	129	(78.7)	**16.70**	**(11.28–24.71)**	**< 0.001**
Cardiovascular Disease – no	1795	(89.3)	80	(48.8)	Ref.		…
Cardiovascular Disease – yes	216	(10.7)	84	(51.2)	**13.64**	**(9.58–19.43)**	**< 0.001**
Obstructive Pulmonary Disease – no	1786	(88.8)	110	(67.1)	Ref.		…
Obstructive Pulmonary Disease – *yes*	225	(11.2)	54	(32.9)	**4.81**	**(3.35–6.91)**	**< 0.001**
Diabetes Mellitus – no	1780	(88.5)	85	(51.8)	Ref.		…
Diabetes Mellitus – yes	231	(11.5)	79	(48.2)	**10.04**	**(7.09–14.23)**	**< 0.001**
No. of Chronic Conditions – no (%)							
0–1	330	(16.4)	115	(70.1)	Ref.		…
≥2	1681	(83.6)	49	(29.9)	**17.81**	**(12.39–25.62)**	**< 0.001**
Complications – no (%)							
Death – no	1965	(97.7)	130	(79.3)	Ref.		…
Death – *yes*	46	(2.3)	34	(20.7)	**39.99**	**(20.23–79.08)**	**< 0.001**

* All cases had been confirmed to have severe acute respiratory syndrome coronavirus 2 (SARS-CoV-2) by polymerase chain reaction (PCR) assay. Of the total cases, gender was unknown in 9 cases. Ethnicity was unknown in 230 cases. Race and ethnic groups were reported by the patient. Percentages may not total 100 because of rounding. Abbreviations: OR, odds ratio; CI, confidence interval; Ref, reference group; RUCCs, rural urban continuum codes.

a.) Obesity was determined by having a most recently recorded body-mass index (BMI) of 30 or more in the last 12 months of data extraction. Of the total cases, 763 had a BMI of ≥30; 978 had a BMI of <30; and 270 had missing data. b.) Smoker was determined by having a past or present history of smoking recorded in the medical record. c.) Absence of a diagnosis recorded in medical record was assumed to mean absence of the chronic condition. d.) Obstructive pulmonary disease was used to indicate a history of asthma and/or chronic obstructive pulmonary disease (COPD). e.) No. of Chronic Conditions was determined by totaling the assessed chronic conditions (hypertension, cardiovascular disease, obstructive pulmonary disease, and/or diabetes mellitus) in each case.

**Table 2:** Association between rurality and clinical characteristics of adult West Virginians with SARS-CoV-2*

	Rural Cases		Urban Cases				
VARIABLES	(N = 692)		(N = 1319)		OR	(95% CI)	*P value*
Age group year – no (%)							
≥ 65	142	(20.5)	188	(14.3)	Ref.		...
40 – 65	256	(37.0)	455	(34.5)	**1.34**	**(1.03–1.75)**	**< 0.001**
18 – <40	294	(42.5)	676	(51.3)	**1.74**	**(1.34–2.25)**	**0.01**
Gender – no (%)							
Male	340	(49.1)	624	(47.3)	Ref.		...
Female	343	(49.5)	695	(52.7)	1.10	(0.92–1.33)	0.29
Race – no (%)							
White	586	(84.7)	1027	(77.9)	Ref.		…
Black	33	(4.8)	92	(7.0)	**1.59**	**(1.06–2.40)**	**0.03**
Other or Unknown	73	(10.6)	200	(15.2)	**1.56**	**(1.17–2.08)**	**<0.001**
Ethnicity – no (%)							
Non-Hispanic	590	(85.3)	1109	(84.0)	Ref.		…
Hispanic	3	(0.4)	79	(6.0)	**14.01**	**(4.40–44.56)**	**<0.001**
Obesity – no (%)							
Obese – no	312	(45.1)	666	(50.5)	Ref.		
Obese – *yes*	258	(37.3)	505	(39.3)	0.92	(0.75–1.12)	0.4
Smoking History – no (%)							
Smoker – no	597	(86.3)	1113	(55.3)	Ref.		..
Smoker – yes	95	(13.7)	206	(15.6)	1.16	(0.89–1.51)	0.26
Chronic Conditions – no (%)							
Hypertension – no	539	(77.9)	1009	(76.5)	Ref.		…
Hypertension – *yes*	153	(22.1)	310	(23.5)	1.08	(0.87–1.35)	0.48
Cardiovascular Disease – no	612	(88.4)	1183	(89.7)	Ref.		…
Cardiovascular Disease – yes	80	(11.6)	136	(10.3)	0.88	(0.66–1.18)	0.39
Obstructive Pulmonary Disease – no	616	(89.0)	1170	(88.7)	Ref.		…
Obstructive Pulmonary Disease – *yes*	76	(11.0)	149	(11.3)	1.03	(0.77–1.38)	0.83
Diabetes Mellitus – no	604	(87.3)	1176	(89.2)	Ref.		…
Diabetes Mellitus – yes	88	(12.7)	143	(10.8)	0.83	(0.63–1.11)	0.21
No. of Chronic Conditions – no (%)							
0–1	574	(83.0)	1107	(83.9)	Ref.		...
≥ 2	118	(17.0)	212	(16.1)	0.93	(0.73–1.19)	0.57
Complications – no (%)							
Hospitalization – no	637	(92.1)	1210	(91.7)	Ref.		…
Hospitalization – yes	55	(8.0)	109	(8.3)	1.04	(0.74–1.46)	0.81
Death – no	672	(97.1)	1293	(98.0)	Ref.		…
Death *– yes*	20	(2.9)	26	(2.0)	0.68	(0.37–1.22)	0.19

* All cases had been confirmed to have severe acute respiratory syndrome coronavirus 2 (SARS-CoV-2) by polymerase chain reaction (PCR) assay. Gender was unknown in 9 cases. Ethnicity was unknown in 230 cases. Race and ethnic groups were reported by the patient. Percentages may not total 100 because of rounding. Abbreviations: OR, odds ratio; CI, confidence interval; Ref, reference group.

a.) Obesity was determined by having a most recently recorded body mass index (BMI) of 30 or more in the last 12 months of data extraction. (763 had a BMI of ≥30; 978 had a BMI of <30; and 270 had missing data). b.) Smoker was determined by having a past or present history of smoking recorded in the medical record. c.) Absence of a diagnosis recorded in medical record was assumed to mean absence of the chronic condition. d.) Obstructive pulmonary disease was used to indicate a history of asthma and/or chronic obstructive pulmonary disease (COPD). e.) No. of Chronic Conditions was determined by totaling the assessed chronic conditions (hypertension, cardiovascular disease, obstructive pulmonary disease, and/or diabetes mellitus) in each case.

**Table 3:** Association between combined race & rurality status and clinical characteristics among adult West Virginians with SARS-CoV-2*

	Urban	Urban	Urban	Rural	Rural	Rural
VARIABLES	White	Black	Other	White	Black	Other
	**OR (95% CI)**					
Gender (Male)	Ref.	0.68 (0.44–1.05)	0.61 (0.45–0.83)	0.91 (0.74–1.11)	0.68 (0.34–1.36)	**0.34 (0.20–0.59)**
Obese	Ref.	0.98 (0.62–1.52)	0.98 (0.67–1.42)	1.07 (0.86–1.33)	1.62 (0.77–3.40)	0.94 (0.41–2.13)
Smoker	Ref.	1.51(0.90–2.53)	**0.54 (0.33–0.89)**	0.89 (0.67–1.18)	1.64 (0.73–3.69)	**0.07 (0.01–0.51)**
Hypertension	Ref.	1.50 (0.95–2.36)	**0.31 (0.19–0.51)**	0.87 (0.69–1.11)	**2.78 (1.38–5.57)**	**0.13 (0.04–0.40)**
Cardiovascular Disease	Ref.	0.69 (0.33–1.47)	**0.15 (0.05–0.41)**	0.99 (0.72–1.35)	1.96 (0.83–4.61)	0.31 (0.10–1.01)
Obstructive Pulmonary Disease	Ref.	1.31 (0.72–2.38)	**0.42 (0.22–0.8)**	0.92 (0.67–1.27)	**2.73 (1.24–6.01)**	**0.10 (0.01–0.73)**
Diabetes Mellitus	Ref.	1.27 (0.68–2.35)	**0.49 (0.27–0.91)**	1.18 (0.87–1.61)	2.07 (0.88–4.88)	0.33 (0.10–1.07)
≥ 2 Chronic Conditions	Ref.	1.38 (0.83–2.31)	**0.25 (0.13–0.47)**	0.99 (0.75–1.29)	**3.04 (1.48–6.22)**	**0.20 (0.06–0.64)**
Hospitalization	Ref.	1.41 (0.73–2.75)	**0.43 (0.21–0.91)**	0.82 (0.56–1.20)	**3.33 (1.46–7.60)**	0.60 (0.22–1.69)
Death	Ref.	0.46 (0.06–3.43)	0.21 (0.03–1.56)	1.25 (0.67–2.34)	2.70 (0.61–11.92)	0.58 (0.08–4.35)

* All cases had been confirmed to have severe acute respiratory syndrome coronavirus 2 (SARS-CoV-2) by polymerase chain reaction (PCR) assay. OR, odds ratio; CI, confidence interval; Ref., reference group.
